# Gravireceptors in eukaryotes—a comparison of case studies on the cellular level

**DOI:** 10.1038/s41526-017-0018-8

**Published:** 2017-04-28

**Authors:** Donat-P. Häder, Markus Braun, Daniela Grimm, Ruth Hemmersbach

**Affiliations:** 1 Erlangen-Nürnberg, Dept. Biol. Neue Str. 9, Emeritus from Friedrich-Alexander Universität, Möhrendorf, 91096 Germany; 2 Gravitational Biology, Universität Bonn, Kirschallee 1, Bonn, 53115 Germany; 3 Department of Biomedicine, Pharmacology, Aarhus University, Aarhus C, DK 8000 Denmark; 4 Institute of Aerospace Medicine, Gravitational Biology, DLR (German Aerospace Center), Cologne, Linder Höhe 51147 Germany

## Abstract

We have selected five evolutionary very different biological systems ranging from unicellular protists via algae and higher plants to human cells showing responses to the gravity vector of the Earth in order to compare their graviperception mechanisms. All these systems use a mass, which may either by a heavy statolith or the whole content of the cell heavier than the surrounding medium to operate on a gravireceptor either by exerting pressure or by pulling on a cytoskeletal element. In many cases the receptor seems to be a mechanosensitive ion channel activated by the gravitational force which allows a gated ion flux across the membrane when activated. This has been identified in many systems to be a calcium current, which in turn activates subsequent elements of the sensory transduction chain, such as calmodulin, which in turn results in the activation of ubiquitous enzymes, gene expression activation or silencing. Naturally, the subsequent responses to the gravity stimulus differ widely between the systems ranging from orientational movement and directed growth to physiological reactions and adaptation to the environmental conditions.

## Introduction

All prokaryotic and eukaryotic organisms are exposed to and respond to an array of physical and chemical stimuli in their environment. Many organisms perceive light at various wavelengths and use this clue to optimize their niche in the habitat.^1^ Other organisms react to chemicals, which may serve as attractant or repellent.^2–4^ This class of chemically-induced behavioral responses includes also reactions to oxygen and other gases.^5, 6^ Several organisms have been found to orient with respect to the magnetic field of the Earth^7^ or to thermal gradients.^8^ Others use electrical signals as cues to orientation movements.^9–11^

Life on our planet has developed under the permanent influence of gravity and all organisms are exposed to this force.^12, 13^ Therefore it can be assumed that most organisms utilize this constant external factor for development and habitat selection. Probably only very small organisms, such as viruses and small bacteria do not perceive and respond to gravity since their behavior is governed by forces of the Brownian motion, which results in a random orientation.^14^

Gravitational forces are perceived by specific receptors activated by either intracellular organelles/structures or by the weight of the complete contents of a cell, which is usually heavier than the surrounding medium (water or air) and thus pressing on the lower cell membrane. There it can be recorded by specific detectors such as mechanosensitive ion channels.^15^ Other options include cytoskeleton elements which pull on membrane structures under the pressure of the cytoplasmic content of the cell as has been proposed for the mechanism of higher plant gravity sensing.^16^

Alternatively, organisms have developed specialized gravisensing organs or organelles which can be found from protists, such as the ciliate *Loxodes*,^17^ algae such as *Euglena* or *Chara* ^1, 18^ to higher plants such as the shoots and roots of *Arabidopsis*.^19^

The knowledge, which is reviewed here is taken from experiments under normal gravity conditions (1 g), increased gravitational stimulation obtained by centrifugation (hypergravity, that means > 1 g) as well as microgravity (<1 g). Limited access to space flights has initiated constructions aiming to achieve microgravity conditions in the Earth-based laboratory, a situation termed “simulated microgravity”.^20^ The idea to alter the influence of gravity is quite old (for review see ref. 21). It is assumed that rotation of a sample randomizes the gravitational force so that the biological systems does no longer perceive gravity and will show a behavior similar to the one seen under real microgravity conditions. To generate simulated microgravity different methods are in use by scientists (cf. review, see ref. 21). Clinostats and random positioning machines (RPM) are common facilities to treat cell cultures, small animals and plants aiming to neutralize the effect of gravity. The principle of a 2D clinostat is that samples are rotated around one axis which is positioned perpendicular to the direction of the gravity vector. If the diameter of the sample is kept in the range of a few mm and the objects are placed in the center of rotation the accelerations induced by rotation are kept minimal. 3D clinostats and RPM are based on the principle that two rotation axes are arranged in a gimbal mount. Rotation speed (3D clinostat) and additionally rotation direction (RPM) are changed at random. Results obtained in all kinds of microgravity simulation experiments have to be critically discussed with respect of possible artifacts. Comparison of data from the different experimental set-ups reveal that microgravity conditions can be achieved with limitations but to some extent in ground-based facilities.^21^

### *Euglena*

The unicellular photosynthetic flagellate *Euglena gracilis* orients itself in its habitat by moving to or away from a light source (positive and negative phototaxis) depending on the irradiance.^1^ In addition, and especially in the absence of light, the cells move with respect to Earth’s gravity vector.^22^ Young cells swim downward in the water column (positive gravitaxis) and older ones upwards (negative gravitaxis). The direction of movement can be altered by the application of heavy metal ions,^23^ increased salinity^24^ or by high irradiance light.^25^ Numerous experiments in real microgravity (on sounding rockets, satellites and the Space Shuttle) documented that the cells are definitively able to respond to gravity rather than with respect to the magnetic field of the Earth.^26^ The threshold for the gravity-induced response is found at ≤0.16 g.^27^ In contrast to an earlier hypothesis, which posited that the cells are passively aligned by a pure buoy mechanism resulting from tail-heavy cells, gravitaxis has been shown to rely on a physiological active gravireceptor. In contrast to organisms, which possess statoliths, in *Euglena* the whole cell body being heavier (up to 1.05 g/ml) than the surrounding medium,^28^ presses on the lower cell membrane. Since the cell rotates around its long axis during forward locomotion, this pressure initiates a modulated signal when it swims horizontally, as the receptors are thought to be located in a distinct position underneath the trailing flagellum.^1^ Calculations taking into account the small volume of the 35 to 65 µm long cell and the small increment of the internal specific density (up to 5 % over the density of the external medium, depending on culture conditions) determine a force of between 0.57 and 1.13 pN exerted by gravity which is at the physical limit but sufficient to account for perception.^29^ In order to verify a valid signal the cells seem to integrate over several cell revolutions.

Using the molecular technology of RNA interference (RNAi)^30^ allowed us to identify the molecular gravireceptor to be a specific TRP (transient receptor potential) channel. TRP channels constitute a large group of proteins involved in photoperception, nociperception, thermal and tactic sensitivity, taste and osmolarity perception and also mechanoperception.^31^ When the protein synthesis of a specific TRP channel was blocked by RNAi, gravitaxis in *Euglena* was inhibited and the effect lasted for up to 30 days.^32^ Photoorientation was not affected by this treatment.

When activated by gravity, the TRP channels allow a passive Ca^2+^ influx into the cell from the outside along a previously established gradient generated by membrane-bound active Ca^2+^ pumps. The Ca^2+^ influx can be visualized using a fluorescent chromophore such as Calcium Crimson.^33^ When these Ca^2+^ pumps are blocked by vanadate or the Ca^2+^ influx through the TPR channel is blocked by gadolinium or the ion gradient across the membrane is broken down by the application of the Ca^2+^ ionophore calcimycin, gravitaxis is inhibited.^34, 35^

After Ca^2+^ has entered the cell by gravitational activation of the TRP channels, it activates a specific calmodulin which binds up to four Ca^2+^ ions. Calmodulins form a protein family involved in many Ca^2+^-mediated cellular processes.^36^ *Euglena* possesses five different calmodulins as shown by gene sequencing; but inhibition of protein synthesis by RNAi of only one of them (CaM.2) effectively blocked gravitaxis.^32^ Using RT-PCR (reverse transcription polymerase chain reaction) confirmed that no mRNA of the blocked calmodulin was transcribed after application of RNAi. The activated calmodulin was found to stimulate an adenylyl cyclase as indicated by inhibitor studies with indomethacine as well as several calmodulin blockers^37^ all of which block gravitaxis in *Euglena*. In contrast, forskulin activates the adenylyl cyclase^38^ and augments gravitaxis. All these results indicate that the activated calmodulin induces the adenylyl cyclase to produce cyclic adenosine monophosphate (cAMP). This has been confirmed during a parabolic sounding rocket experiment.^39^ cAMP is also involved in the photoperception mechanism in *Euglena* ^40^ and in the gravitactic orientation of the ciliate *Paramecium*.^41^

The produced cAMP finally activates a protein kinase A (PKA), which in turn is thought to modulate the beating pattern of the flagella and thus to cause a reorientation of the cellular swimming path during gravitaxis. Staurosporine, an effective inhibitor of protein kinases,^42^ blocked negative gravitaxis in *Euglena*, but inversed it into a positive gravitaxis after extended exposure times to the inhibitor.^43^ Using degenerated primers revealed five isoforms of PKA (PK.1–PK.5), and their full sequences were revealed by RACE-PCR (rapid amplification of cDNA-ends with polymerase chain reaction). Only RNAi of PK.2 effectively blocked gravitaxis up to several weeks, while blocking the other isoforms had no effect.^43^

**Fig. 1:**
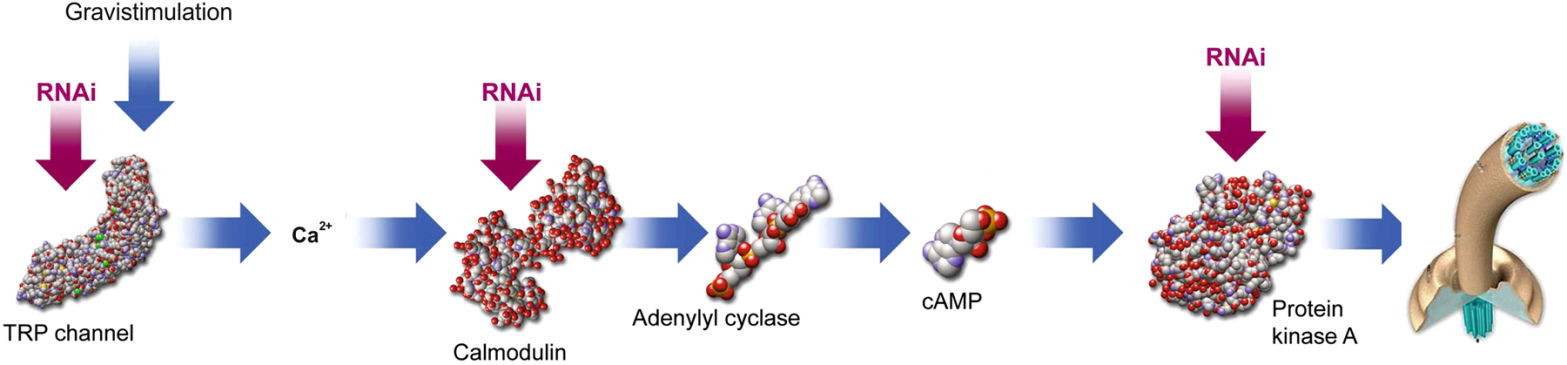
Schematic model of the gravitactic signal transduction chain in *Euglena gracilis* (for details, see text)

Figure 1 shows a schematic description of the gravitactic signal transduction chain in *Euglena gracilis*. During horizontal swimming the cell content exerts a pressure onto the lower membrane resulting in a modulated signal in the TRP channels, located under the trailing membrane, which allow a gated Ca^2+^ influx into the cell. Ca^2+^ activates a specific calmodulin, which in turn induces a likewise specific adenylyl cyclase to produce cAMP. This activates an also specific phosphokinase A believed to finally cause a reactivation of the flagellar beating pattern resulting in the reorientation of the swimming path during gravitaxis of *Euglena*.

### *Loxodes*

Is the graviperception mechanism revealed in *Euglena* a universal mechanism in other protists? To answer this question, we take a look at ciliates, which are considered the most evolved protozoans, possessing cilia for locomotion. The tactic responses to diverse external stimuli are best known in *Paramecium*. Gravity plays a key role for its orientation, especially in the absence of other cues, in order to reach optimal living conditions. Negative gravitaxis under terrestrial conditions and loss of gravitaxis in real and simulated microgravity have extensively been studied, and a threshold for gravitaxis on the order of 0.3 g has been determined by using a centrifuge microscope in space.^44^ Like *Euglena*, *Paramecium* (around four times larger than *Euglena*) uses the heavy cellular content to detect gravity by a physiological mechanism. It is assumed that the pressure of the cell mass activates mechanosensitive ion channels in the outer membrane. A polar distribution of mechanosensitive calcium and potassium ion channels, which is known from electrophysiological studies, triggers either de-polarisation or hyperpolarisation of the cell membrane during downward or upward swimming. Intracellular electrophysiological experiments on the highly mechanosensitive ciliate *Stylonychia mytilus* revealed a true gravireceptor potential. Membrane potential changes of 4 mV during cell reorientation with respect to the gravity vector support the statocyst hypothesis.^45^

In contrast, another ciliate, *Loxodes*, uses a different gravisensing mechanisms. It preferentially glides along solid surfaces (sediment), however increasingly low oxygen concentrations induce *Loxodes* to swim vertically upwards in the water column, whereas high oxygen induces positive gravitaxis in this microaerophilic organism.^46^ In real and simulated microgravity, *Loxodes* loses its orientation behavior and swims in random directions. The threshold for gravitaxis was found at 0.16 g.^44^

**Fig. 2:**
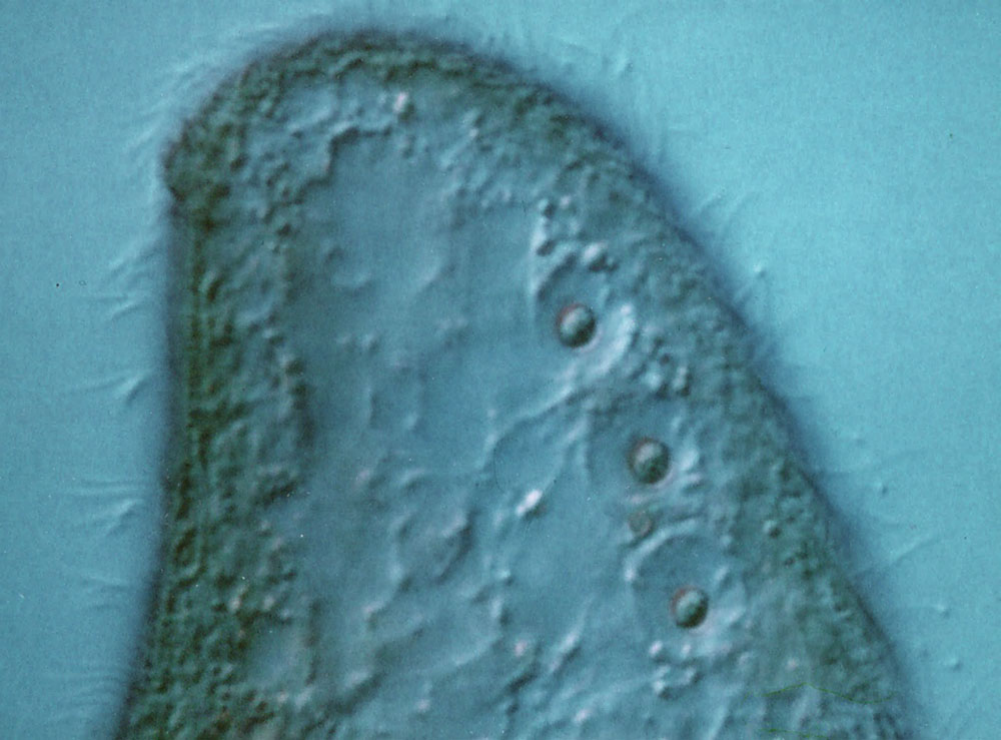
Müller organelles (3) acting as cellular gravisensors in *L. striatus*, containing a barium sulfate granulum (3 µm diameter) fixed to a microtubular structure (courtesy N. Rieder)

While *Euglena* and *Paramecium* lose gravitaxis when the density of the medium equals the cellular density and thus neutralizes a density-dependent pressure gradient via the cell membrane, *Loxodes* still performs gravitaxis under such conditions. This clearly indicates the existence of a statolith activating an intracellular gravisensor, working independently from the density around the cell.^47^ *Loxodes striatus* possesses 3–4 so-called Müller organelles per cell^48^ which bear all characteristics of a true gravisenor. A heavy mass is represented by a barium sulfate body within a vacuole attached to a ciliary stick (Fig. 2). Changes in spatial orientation of the cell and consequently the statolith trigger changes in the membrane potential and, in turn, ciliary activity and cell reorientation. Micro-chirurgical destruction of the connection of the statolith to the ciliary stick by means of a laser beam resulted in complete loss of gravitaxis proving the function of the Müller organelles as cellular gravireceptors.^49^

### *Chara* rhizoids and protonemata

Having found two different principles of graviperception in unicellular protists poses the question of how multicellular organisms detect gravity. Concentrating first on algae, gravireceptors and plant gravitropic signaling has been intensively studied in tip-growing and translucent rhizoids and protonemata of the Chlorophyte *Chara*. Positively gravitropic (downward growing) rhizoids anchor the algal thallus in the sediment, whereas protonemata, morphologically almost identical, respond negatively gravitropic growing upward in darkness (in case they are buried in the sediment). As soon as they grow out into the light they restore the complexly organized green algal thallus. The complete gravitropic signal transduction and response pathways in both cell types are very short, limited to the apical region of a single cell. Both graviresponses are initiated by a microscopically easy to observe gravity-mediated sedimentation of statoliths filled with BaSO_4_ crystals; for review see ref. 50.

In tip-downward growing rhizoids, the statoliths are actively kept in a dynamically stable position 10–35 µm above the tip. By exerting net-basipetal forces the actomyosin system prevents statoliths from settling into the tip, while in tip-upward growing protonemata, actomyosin prevents statoliths from sedimenting towards the cell base by acting net-acropetally.^51^ Experiments in microgravity and in simulated weightlessness, using clinorotation, have provided clear evidence for the complex and well-orchestrated actomyosin forces which both cell types use to regulate statolith positioning,^51^ compensating the gravitational force and thus keeping statoliths in a precisely controlled dynamic position close to the tip. Only in this position they are able to fulfill their function as gravity susceptors initiating gravity sensing.

Upon gravistimulation, statoliths do not simply follow the gravity vector and sediment onto the lower cell flank; in fact, actomyosin forces direct sedimenting statoliths to specific gravisensitive regions of the plasma membrane. These are the only locations, where the gravitropic signaling cascade is elicited triggering the reorientation of the growth direction; see also ref. 51.

Microgravity experiments^52^ and optical laser tweezers experiments have been performed to characterize the complexly arranged actomyosin forces that regulate statolith movements in both rhizoids and protonemata. The laser tweezers force needed to move statoliths towards the apex is much larger than the force required to pull statoliths towards the flank. During two sounding rocket flights (MAXUS 3 und MAXUS 5) lateral centrifugal forces in a range of 0.1 g were sufficient to trigger a movement of statoliths towards the membrane-bound gravireceptors. From these results, the molecular forces acting on a single statolith in lateral direction were determined to be on the order of 2 × 10^−14^ N.^53^

Gravistimulation of tip-upward growing protonemata causes an actin-mediated acropetal displacement of sedimenting statoliths into the apical dome independent of the gravistimulation angle, where they settle onto the gravisensitive plasma membrane which, in protonemata, is a small area very close to the tip, 5–10 µm behind the tip.^51^

Although the nature of the gravisensor molecules in rhizoids and protonemata has not yet been revealed, there is clear experimental evidence that statoliths need to fully sediment and touch the specific membrane areas in both cell types in order to trigger graviperception and to induce the gravitropic signaling cascade.^51^ Lateral movements of statoliths, which do not lead to a contact with the plasma membrane, fail to induce a curvature response. Recently, experiments have been performed during parabolic flights (PFs) on board of the A300 Zero-G aircraft to elucidate the mechanism of gravireceptor activation in characean rhizoids.^53^

During the phases of microgravity statoliths were weightless but still able to activate the membrane-bound gravireceptors as long as they remained in contact with the plasma membrane. Thus, not pressure exerted by the weight of statoliths but contact is required for gravireceptor activation. Accordingly, increasing the weight of sedimented statoliths by lateral centrifugation in ground control experiments did not enhance the gravitropic response and interrupting the contact of statoliths with the plasma membrane by inverting gravistimulated cells terminated graviperception.^53^ However, the components on the statoliths’ surface for the interaction of statoliths with membrane-bound receptors are still unknown.

The actomyosin system that plays a crucial role in the activation of gravireceptors is also an essential component of the tip-growth and graviresponse mechanisms in both cell types. The Spitzenkörper in these cell types is a complex tip-growth generating structure comprised of a central aggregation of endoplasmic reticulum and, among others, vesicles which deliver cell-wall material towards the apical plasma membrane. The integrity and function of the Spitzenkörper is the result of the concerted action of actin and numerous actin-binding proteins.^54^

The smooth downward curvature response of a rhizoid, best described as ‘bending by bowing’, is the result of reduced growth rates of the lower subapical cell flank. In this area, exocytosis of cell-wall material is locally inhibited due to statolith-induced activation of the gravisensor, which was shown to result in a drastically reduced concentration of cytoplasmic calcium. Considering these results, it is tempting to suggest that statoliths touching the cell membrane triggers a local inhibition of calcium channels which is the opposite of the gravity-induced opening of calcium channels in *Euglena*. The Spitzenkörper itself always remains in a fixed position in the center of the apical dome and, thus, the center of maximal growth at the cell tip is not affected during the positive graviresponse in rhizoids.

The negative graviresponse in protonema was described as ‘bending by bulging’^55^ referring to the bulge that appears on the upper cell flank indicating the drastic upward shift of cell growth. The Spitzenkörper and, in consequence, also the center of maximal growth is shifted upward upon gravistimulation of protonemata by intruding statoliths.

There are indications that the specific properties of the actin cytoskeleton which are responsible for the Spitzenkörper anchorage are dependent on calcium. This is strongly supported by fluorescence imaging demonstrating a drastic shift of the steep tip-high calcium gradient and putative calcium channels towards the upper flank during initiation of the graviresponse in protonemata, but not in rhizoids.^56^ The asymmetric influx of calcium might mediate the repositioning of the Spitzenkörper and the growth center by differentially regulating the myosin-mediated anchorage or the activity of actin-associated proteins along the shifting calcium gradient.^56^

### *Arabidopsis* roots

**Fig. 3:**
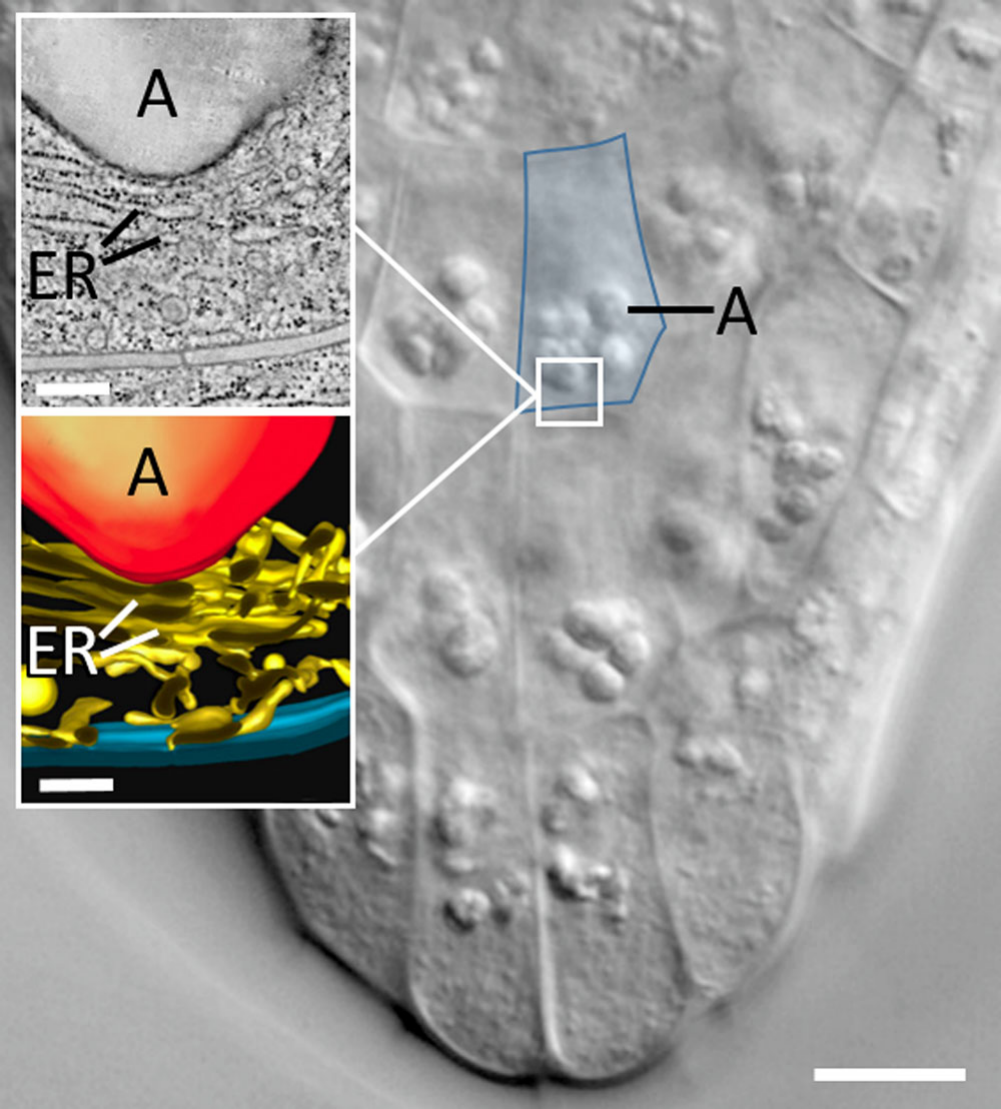
Differential interference contrast micrograph of a root cap of *A. thaliana* showing central columella cells with sedimented amyloplasts; a single columella cell is outlined in *blue*. Bar = 10 µm. *Insets* depict a tomographic slice image and a tomographic model of the lower part of an amyloplast (**a**) deforming tubules and cisternae of the endoplasmic reticulum network (ER) in the lower part of a columella cell of *Medicago sativa*. Bars = 300 nm. Modified after.^60^

We now move from multicellular algae to the more complex higher plants. Primary shoots of higher plants grow vertically upward and primary roots downward guided by gravitropism.^57^ Lateral branches and roots maintain a growth direction at specific angles to the gravitational vector of the Earth.^58^ Specialized cells like the statocytes in roots and shoots of higher plants allow starch-filled amyloplasts to sediment in the direction of gravity.^59^ The sedimenting amyloplasts, called statoliths, serve as susceptors which transform the physical effect of gravity—gravity-directed sedimentation - into a physiological signal by exerting pressure onto an underlying sensing mechanism such as gravireceptor molecules in the endoplasmatic reticulum (ER) under the influence of gravity.^60^ Convincing evidence in support of the starch-statolith hypothesis came from studies in which high-gradient-magnetic fields were used to displace amyloplasts in vertically oriented roots and shoots.^61^ Curvature responses were exclusively induced by displacing statoliths without changing the gravity vector with respect to the plant organ. Mutants with reduced or missing starch in the amyloplasts were found to show a retarded or weaker positive gravitropism^62^ indicating that the presence of starch in amyloplasts in root cells may not be an essential prerequisite for positive gravitropism; but its presence enhances the response.^63^ Recent research has partially revealed the molecular mechanism of gravitropism in *Arabidopsis thaliana*.^64, 65^ The root statenchyma, the so-called columella, is embedded in the root cap below the apical root meristem (Fig. 3). The inner cells of the second row of columella cells are most important for gravitropism; this can be shown by ablating these cells with a laser beam, while the cells in the root cap hardly contribute to the orientational mechanism.^66^ The columella cells contain starch grains, which rest on the accumulation of ER cisternae in the lower part of the cells, whereas the nucleus resides at the top. High-resolution video has demonstrated the movement of amyloplasts within the statocytes.^67^ Sedimenting amyloplasts seem to deform the ER membrane which is in contrast to *Chara* rhizoids and protonemata in which the sedimenting statoliths only have to touch the membrane but not to exert a force on it. In higher plant roots the depression of the statoliths onto the ER might induce a release of calcium from this intracellular reservoir.^68^ However, the involvement of the ER in the gravitropic response is still hypothetical. The statoliths do not just passively sediment, but show continuous, in shoot cells sometimes, saltatory movements.^69^ This seems to be brought about by the actomyosin system, which interacts with the amyloplasts also during sedimentation.^68^

Several hypotheses assume that the sedimenting amyloplasts exert a force on the F-actin filaments, which might activate mechanosensitive channels and, thus, could translate the mechanical signal into a biochemical signal. However, application of actin inhibitors such as latrunculin B^70^ results in enhanced gravitropism, rather than inhibiting it, indicating that actin might operate as a negative regulator for the response.^59^ The nature of the gravireceptor and the mechanism of gravity perception are far from being disentangled. Even the vacuole has been suggested to play a role at least in shoot gravitropism in *Arabidopsis*.^63^

Since gravity sensing in roots is limited to statocytes in the root cap and the response is facilitated through differential root flank growth further up in the elongation zone, a transduction chain must connect the statocytes and the elongating cells in the growth zone.^71^ The plant hormone auxin plays a key role in cell growth in higher plants. Auxin is known to be produced in the shoot apex and transported downward to the roots mediated by numerous influx and efflux carriers. In the columella cells at the root tip, auxin is redirected laterally and then transported upward along the root flanks to the elongation zone. Upon gravistimulation, statolith sedimentation and gravireceptor activation most likely at the ER membranes leads to lateral polarization of the statocytes, which involve cytoplasmic alkalinization and an apoplastic acidification^72^ and relocation of the auxin efflux facilitator PIN3 towards the lower flank of the statocytes.^73^ As a result, the auxin flux along the lower flank of the root is increased leading to gravitropic curvature by differential growth.

Molecular studies have revealed that the proteins ARL2 and ARG1, which are located in endomembranes, are required for lateral redistribution of auxin and PIN3 relocation upon gravistimulation. Mutations in the two paralogous *Arabidopsis* genes, encoding J-domain proteins, lead to reduced root and shoot gravitropism. ARL2 and ARG1 are candidates for components of the gravitropic signaling pathway that mediate changes in the activity and/or localization of proteins which may contribute to gravisensing-related processes like cytoplasmic alkalinization and auxin redistribution.

### Human cells

Finally we will look into human cells to cover a wide range of graviresponsive eukaryotes including animals and plants.

Data from real and simulated microgravity experiments had shown that human T lymphocytes activation is clearly inhibited demonstrating that microgravity affects early cell activation events.^74^ Lymphoblast lung U937 cells exposed to real and simulated microgravity after loading them with a radio-labeled phorbol ester showed a significant translocation of protein kinase C (PKC) at all *g* levels. These data indicated that the sensitivity of the PKC to this stimulus provides useful means for measuring the effects of altered gravity levels during early cell activation events.^74^ The influence of gravity on the interleukin (IL) IL-2 and IL-2-R-alpha expression^75^ in human leukocytes was demonstrated as well. Another study also identified PKA to be a gravity-dependent regulator with respect to the loss of T-cell activation in microgravity. The nuclear factor kappa-light-chain-enhancer of activated B-cells (NF-kB), the transcription factors AP-1 and the cAMP response element-binding protein are all regulated by PKA, and their gene expression was altered after exposure to microgravity.^76^

Taken these data together, a variety of transcription factors are obviously involved in gravisensitivity of human lymphocytes, such as NF-kB, PKC and PKA.

Short-term microgravity exposure (22 s) during PF maneuvers induced early cytoskeletal changes and altered gene expression in endothelial cells (EC).^77^ Several gravisensitive signaling elements, such as AMP-activated protein kinase alpha 1 and integrins are involved in the reaction of EC to altered gravity conditions.^77^ Chondrocytes cultured on a random positioning machine (RPM) exhibited early cytoskeletal changes^78^ as shown by an increased expression of several genes of cytoskeletal components (beta-tubulin, vimentin) after 30 min of exposure. After 4 h, disruptions in the vimentin network were detected. After 16 h, however, the chondrocytes reorganized their cytoskeleton demonstrating cellular adaptation capacities. However, the transforming growth factor beta 1 (TGF-β_1)_ gene and protein were elevated for 24 h in microgravity.^78^ Human chondrocytes cultured during a PF mission showed no changes after the 1st parabola, but disruptions of the β-tubulin, vimentin, and cytokeratin networks after the 31st parabola. Even after 31 parabolas no changes were found in F-actin.^79^ In contrast to low-differentiated thyroid cancer cells, human EC and chondrocytes only exerted moderate cytoskeletal alterations.^79^ The significantly elevated expression of the bone morphogenetic protein 2, TGF-β_1_ and the transcription factor SOX9 in human chondrocytes may have protective effects on the cytoskeleton of chondrocytes.

Real microgravity-induced cytoskeleton and focal adhesion alterations in bone cells are the two major mechanosensitive responses. The cytoskeleton responds to changes in the mechanical environment because it is connected to the extracellular matrix (ECM) through focal adhesions. Exposure of osteoblasts to microgravity impaired their cytoskeleton stability and reduced cellular tension, as well as focal adhesion formation and stability.^80^ Cancer cells are also sensitive to mechanical forces and the microgravity environment induced a specific alteration of the cytoskeleton. Human Michigan Cancer Foundation 7 breast cancer cells exhibited alterations of the cytoskeleton after real and simulated microgravity exposure.^81, 82^ During a PF changes of F-actin were detected in human thyroid tumor (ML1) cells even after the 1st parabola.^83^ A similar result was observed in EC which were fixed during the flight.^77^ With the help of the compact fluorescence microscope (FLUMIAS) for fast live-cell imaging under real microgravity it is now possible to investigate the cytoskeletal changes in space.^84^ During the TEXUS-52 sounding rocket flight, the FLUMIAS microscope revealed significant alterations of F-actin related to real microgravity.^84^

Human cells can react in vitro to mechanical unloading in different ways. However, the question arises, how are they able to sense the rather weak changes in force? Ever since Rijken et al. found significant alterations of the cytoskeleton in human A431 cells during a TEXUS flight in 1991,^85, 86^ the cytoskeleton is a hot candidate of transmitting mechanical unloading from the cells’ environment. How the cells manage to transform the mechanical signal into a biochemical one is still an unresolved topic. However, an increasing number of data support the tensegrity model hypothesis proposed by Ingber.^87^ The tensegrity model claims that cells are hardwired by the different parts of the cytoskeleton, which are connected to discrete cell adhesions. By this, cells are spanned open and are under continuous tension comparable to tent poles fastening a tent. The focal adhesion points are connected to the ECM. In summary, there is a balance of forces between ECM, adhesion points and the cytoskeleton at normal gravity conditions. Therefore, an imbalance of adhesion and cytoskeleton would result in a change of cell shape and has a direct impact on signaling cascades and downstream transcription events.^87^ This theory is supported by data of cytoskeletal changes in different cell types after short-term exposure to real or simulated microgravity.^88^ Fixation of cells after 22 s of microgravity revealed that actin fibers and/or microtubules were localized close to the nucleus while losing their distinct polarization.^77, 79, 83^ These findings are in concert with significant gene expression changes after 22 s of real microgravity.

However, artifact induction during fixation could not be excluded until Corydon et al. first investigated life-act GFP transfected thyroid cancer cells during a PF (TEXUS 52).^84^ Live imaging of the cells in microgravity revealed an instant rearrangement of actin filaments and a rapid change of cell shape.

Finally, these experiments further increase the evidence of a direct correlation of the cytoskeletal rearrangements upon microgravity and transcription alterations and strongly suggest the interaction of the ECM, adhesion and connected cytoskeleton to be the basis of gravisensing in human cells.

### Common principles or differences in graviperception between evolutionary diverse organisms

Gravity is a unique permanent environmental factor on Earth. Biological systems have developed under this constant condition and adapted themselves to the stimulus. Like other sensors types, which collect information on environmental factors, such as oxygen, chemicals, and light, gravity sensors are coupled to signaling pathways, which can be divided into the steps of perception, transduction, amplification and response.^89, 90^

Dedicated experimental hardware allows experimentation in real and simulated microgravity, which in comparison with ground-based studies, elucidate the principles of gravity sensing and signaling and interaction in the cellular and organismic functionality. Our comparison of evolutionary very diverse systems demonstrates common principles and reveals major components which are indispensible to sense gravity. In all studied systems an entity with a distinct mass perceives the force of gravity and conveys this signal to a suitable receptor. The mass can be either a heavy statolith as in the examples of *Chara* and *Loxodes* ^49, 51^ or it can be the whole cell content which is heavier than the outside medium as in the cases of *Euglena*, *Paramecium* and human cells.^1, 84, 91^ In statocytes of higher plants heavy amyloplasts have been identified as statoliths which need to be displaced by gravity,^67^ but in their absence the cellular content can play the role of a heavy mass exerting a force on the gravireceptor.^92^

The next step is to identify the receptor, which senses the force of the heavy mass being either a statolith or the whole cell content. In the case of *Euglena* mechanosensitive ion channels have been identified as the receptors in the form of dedicated TRP proteins.^32^ Other members of the large TRP protein family have been found to be involved in mechanosensitivity.^31^ E.g. the TRPC1 channel has been found to form a stretch-sensitive ion channel in vertebrates.^93^ Ion channels are also involved in graviperception in ciliates.^45, 94^ In contrast, in *Chara* the statolith does not have to exert a force on the gravisensitive region of the plasma membrane. It is sufficient to establish a contact to induce the response.^53^

In statocytes of higher plants amyloplasts have been found to press onto and deform the membranes of the underlying endoplasmatic reticulum.^60, 95^ This pressure seems to result in a release of Ca^2+^ from the ER vesicles into the cytoplasm. Mechanosensitive ion channels could be involved but have not yet been identified. Whether or not the gravitropism in amyloplast-deficient mutants is based on a different mechanism still has to be elucidated. It has been proposed that in that case the (heavy) cell content could exert a force on the cytoplasmic membrane by interacting with cytoskeletal filaments.^96^ This also seems to be the case in human cells which do not possess statoliths. Here the available results indicate that the cell content exerts a force on the cytoskeleton filaments linked to the plasmamembrane.^79^ It could be speculated that this force might operate mechanosensitive channels.

Altered microgravity conditions induce numerous effects on the plasma membrane in human cells, and apoptosis (membrane blebbing) occurred in several cell types.^97–99^ Changes in microvilli and lamellipodia were detected in viable thyroid cancer cells in real microgravity.^84^ Cells revealed an altered composition of laminin, and collagen IV, both major components of the basal laminae and important for the maintenance and survival of tissues.^78, 100–102^ Both ECM proteins are efficiently inducing the polarization of epithelial cells.^103–105^ Microgravity altered different types of membrane structures, such as the caveolae,^106^ focal adhesions,^107, 108^ like vinculin^109, 110^ or cell junctions, which are responsible for cell adhesion and communication. Human cells may sense gravity changes via signals transmitted across transmembrane adhesion receptors linking to the cytoskeleton, the ECM and to other cells (e.g., integrins, cadherins, selectins).^87^ Various transmembrane proteins like growth factor receptors, adhesion proteins or ion channels are associated with the sub-membranous system of actin filaments and control their force-generating capacity. The actin cytoskeleton, which was found to be rearranged in real microgravity,^84, 110^ is connected to several membrane proteins, influencing polarity, cell adhesion, migration and the response to extracellular signals. It is known that the cytoskeleton, adhesion molecules and ECM form a dynamic network interacting with signaling molecules which showed an adaptive response to changing gravity conditions of PFs.^83^ Cells attach to the ECM containing adhesive proteins that bind to transmembrane regulatory proteins (e.g., integrins). They interact with the cytoskeleton and the cytoskeleton ultimately connects to the cell nucleus rendering it possible that an external signal such as a change in gravity conditions leads to the cytoskeleton-mediated activation of regulatory proteins in the cell membrane and influences directly the regulation of gene expression.

Ingber has published the important concept of ‘tensegrity’ (i.e., tensional integrity), a tension-dependent form of cytoskeleton-based cellular structure stabilizing the cellular form.^87^ When altered gravity conditions affect the cells, microtubules rapidly reorient themselves and actin stress fibers increase in density in order to reinforce their mechanical strength. Accordingly, changes in the actin and tubulin cytoskeleton and shedding of membrane receptors accompanied by changes in cell shape, cell detachment, apoptosis, changes in growth behavior, differentiation, and migration have been detected in real microgravity and on Earth using various µg-simulation devices.^84, 86, 95–103, 111, 112^

While it has not been proven for all studied systems, a working hypothesis could be that the pressure of a statolith or the heavy cell content activates mechanosensitive channel either by direct pressure or by pulling on cytoskeleton elements. These membrane proteins could allow an ion transport when activated which in some cases has been found to consist of calcium ions. At least in the case of *Euglena* the gated calcium activates calmodulin,^32, 113^ which is a universal regulatory protein in many prokaryotic and eukaryotic taxa.^36, 114^ The influx of Ca^2+^ changes the membrane potential as has been found in ciliates and flagellates, which might be a subsequent step in the sensory transduction chain being responsible for signal amplification.

While the primary receptors and mechanisms of graviperception are remarkably similar in evolutionary very diverse systems, they differ widely in their gravitational responses. The visible reactions range from movement reorientation to directed growth responses, from physiological reactions to gene activation or silencing. Recent results showing fast gravity-related changes in the fluidity of cell membranes and consequently postulated gravity-dependent functional changes of membrane-integrated proteins^115^ as well as very fast changes of the phosphorylation status of proteins observed after a few seconds of exposure to microgravity (unpublished results) indicate other cellular elements to be involved in the signal transduction and amplification of gravitational stimuli. We conclude from our review that at least all eukaryotic organisms are able to sense gravity.

Recommendations for future research can be derived from this review for the analysis of graviresponsive organisms in which no molecular gravireceptor has been identified yet. One obvious option is to look for mechanosensitive ion channels and gated ion transport. This involves molecular genetic studies searching for e.g. ubiquitous TRP channels, quantification of ion transport such as calcium using fluorophores and identifying subsequent steps such as activation of signaling proteins and enzymes.
